# Multispecies reconstructions uncover widespread conservation, and lineage-specific elaborations in eukaryotic mRNA metabolism

**DOI:** 10.1371/journal.pone.0192633

**Published:** 2018-03-21

**Authors:** Bridget P. Bannerman, Susanne Kramer, Richard G. Dorrell, Mark Carrington

**Affiliations:** 1 Department of Biochemistry, University of Cambridge, Cambridge, United Kingdom; 2 Biozentrum, Lehrstuhl für Zell-und Entwicklungsbiologie, Universität Würzburg, Am Hubland, Würzburg, Germany; 3 Institute of Biology, École Normale Supérieure, PSL Research University, Paris, France; Instituto Butantan, BRAZIL

## Abstract

The degree of conservation and evolution of cytoplasmic mRNA metabolism pathways across the eukaryotes remains incompletely resolved. In this study, we describe a comprehensive genome and transcriptome-wide analysis of proteins involved in mRNA maturation, translation, and mRNA decay across representative organisms from the six eukaryotic super-groups. We demonstrate that eukaryotes share common pathways for mRNA metabolism that were almost certainly present in the last eukaryotic common ancestor, and show for the first time a correlation between intron density and a selective absence of some Exon Junction Complex (EJC) components in eukaryotes. In addition, we identify pathways that have diversified in individual lineages, with a specific focus on the unique gene gains and losses in members of the Excavata and SAR groups that contribute to their unique gene expression pathways compared to other organisms.

## Introduction

The eukaryotic tree of life is a complex, elaborate, and beautiful structure. Multicellular lineages such as animals and plants, and model microorganisms such as yeast, only form a small minority of its branches, with the majority of the eukaryotic tree consisting of protists [1]. These other eukaryotic branches are highly diverse in terms of cell organisation, fundamental biochemistry, and life strategy. For example, pathogenicity and parasitism has independently evolved many times, on disparate branches of the eukaryotic tree. These distantly related parasitic lineages perhaps most notably, in terms of human impact, include: the pathogenic apicomplexans such as *Plasmodium*, the causative agent of malaria [2]; the kinetoplastids including *Trypanosoma brucei*, the causative agent of sleeping sickness [3]; and the oomycetes which are important crop pathogens, including potato late blight [4].

Over the last three decades, phylogenetic and latterly phylogenomic studies have largely resolved the major evolutionary relationships between different eukaryotic organisms, leaving six major super-groups, incorporating all but a small minority of eukaryotic lineages [1, 5]. These are: Opisthokonta containing animals and fungi, Amoebozoa containing most amoebae, Excavata containing many anaerobic lineages, as well as kinetoplastids, Archaeplastida containing plants, green algae and red algae, the ‘SAR clade’ containing diatoms, oomycetes and apicomplexans and the ‘CCTH clade’ containing chalk-forming haptophyte algae [1, 5]. These groups are largely verified to each be monophyletic, although significant debate still exists over the inclusion of certain lineages within Excavata [6, 7] and over the unity of the CCTH clade [8, 9]. The position of the root of the eukaryotic tree also remains debated, although it is likely to be positioned either between the Excavata and a monophyletic group of Amoebozoa and Opisthokonta, or between Excavata and a monophyletic group of Archaeplastida, CCTH and SAR [6, 10].

The enhanced higher-order resolution of the eukaryotic tree has enabled unprecedented insight into the features that are broadly conserved, hence were probably present in the last eukaryotic common ancestor (LECA). It is now well established that LECA possessed mitochondria [11, 12], a complex nucleus and microtubule organising centre [13] and an elaborate endomembrane system [14]. Reconstructing the cellular processes associated with LECA may help resolve the specific changes to cell biology that have accompanied the origins of major eukaryotic clades and life strategies, such as parasitism. Free-living eukaryotes that are closely related to parasitic species such as the chromerid algae *Chromera velia* and *Vitrella brassicaformis* [15, 16] which are closely related to parasitic apicomplexans, or the non-parasitic bodonids [17] and heterolobosean protist *Naegleria gruberi* [18], which are related to parasitic kinetoplastids, may provide further insights into the processes that originate, are lost, or are significantly altered during the transition to parasitism.

Here, we elaborate on the eukaryote-wide evolution of an important component of the cell biology of LECA, the mRNA metabolism. Gene expression in eukaryotic nuclei begins with mRNA transcription and progresses through mRNA maturation, export of the transcript from the nucleus, translation to produce the gene product and, finally, mRNA decay. We focus on three different aspects of this pathway: i) the exon junction complex that is involved in mRNA quality control; ii) the translation initiation complex with its binding partners’ poly(A) binding protein and Dhh1; and iii) 5’-3’ mRNA decay.

The central role of the exon junction complex (EJC) is to mark the splice sites in eukaryotic pre-mRNA transcripts. It thus provides a memory of the splicing process that could for example be used to detect premature stop codons and induce nonsense mediated decay of faulty transcripts [19]. Three core components of the EJC; Magoh, Y14 and eIF4AIII have been identified amongst eukaryotic super-groups whilst a fourth component, MLN51 was identified only in animals [19, 20]. Previous studies have shown that the core EJC proteins are conserved in the Archaeplastida and Opisthokonta groups [20, 21 22, 23, 24] except in *S*. *cerevisiae* where the only component of the EJC identified was eIF4AIII (encoded by the *FAL1* gene) [25]. However, it is not known whether the EJC is conserved in other eukaryotes.

During translation initiation, the mRNA is bound by eIF4F, a complex containing the RNA helicase eIF4AI, the cap-binding protein eIF4E, and the adaptor protein eIF4G to form a closed circle through interaction of eIF4G with Poly(A) Binding Protein, PABP [26, 27]. The eIF4F complex binds to the small subunit of the ribosome and scans to the initiating AUG codon where the large ribosomal subunit joins. Many of the factors involved in translation initiation have previously been shown to have undergone duplication events in specific eukaryotic groups, and in parasitic members of the SAR clade and Excavata. For example, parasitic kinetoplastids possess up to six eIF4E homologues compared to one to two in most Archaeplastida and Opisthokonta groups [28, 29] and five eIF4G isoforms compared to two to three in Archaeplastida and Opisthokonta [28]. Several of these novel duplicate kinetoplastid isoforms have been shown to have specific and complementary functions, such as *Leishmania* eIF4E6, which interacts with eIF4G5 [29], and *Trypanosoma brucei* eIF4E4, which binds to eIF4G3 [28]. The broader overall interactions, and evolutionary histories of these different duplicated subunits remain to be determined.

Finally, mRNAs are turned over in eukaryotic cells. In Opisthokonta, there are two major cytosolic mRNA degradation pathways. Both start with deadenylylation by the Ccr4/Caf1/Not complex that acts in conjunction with several other subunits (Caf4, Caf16, Caf40 and Caf130p, Not1 to Not5), and a second deadenylase complex, Pan2p and Pan3p; [30, 31]. After deadenylylation, the cap structure is hydrolysed, by the decapping complex Dcp1 and Dcp2, which is stimulated by Edc1-3 in yeast [32, 33]. The evolutionary distributions of many of these subunits across the eukaryotes remain unresolved, although not all are universally conserved. For example, Edc3 is conserved in Opisthokonta, while Edc1p and Edc2p are found only in yeast such as *Saccharomyces cerevisiae* [34]. Neither of the decapping subunits Dcp1 or Dcp2 has been found in trypanosomes; the recently reported Dcp2-like enzyme has very poor *in vitro* decapping activity towards a mature trypanosome cap and is very unlikely to be the functional homologue to Dcp2 [35]. Instead, trypanosomes use an ApaH like phosphatase as their major decapping enzyme, which is unrelated to Dcp2 [36]. Following deadenylylation and decapping, the mRNA is degraded in the 5’ to 3’ direction by the exoribonucleases Xrn1 and Rat1p [37, 38]. Xrn1 and Rat1p are functionally redundant subunits, which in yeast respectively function in the cytoplasm and the nucleus [39]. mRNA may also be degraded in the 3’ to 5’ direction by the exosome complex.

The evolutionary distribution of exosome subunits across the eukaryotes has already been well studied [40, 41], with evidence for lineage-specific duplications and reductions in the number of genes encoding exosome subunits [41, 42, 43] and will not be discussed in further detail. However, the distribution and function of other mRNA turnover complexes across the eukaryotes remains poorly resolved. For example, it has previously been shown that a modified Not complex consisting of multiple subunits (Not1, Not2, Not3, Not5, Not9 and Not10) exists in *Trypanosoma brucei*, of which Not10, Not9, Not1 and Caf1 have previously been shown to interact directly between one another [44]. In addition, trypanosomes possess four Xrn homologues (a cytoplasmic Xrn1 homologue, XRNA, a nuclear Rat1p homologue, XRND; and two further homologues, XRNB and XRNC, which have unknown localizations and functions [45]. However, to date it is not known if expanded or reduced 5’ decay complex is present in other organisms.

In this study, we compare sequence data from a taxonomically broad range of eukaryotic genome and transcriptome libraries. We determine the conservation of the EJC, translation initiation factors and proteins involved in mRNA degradation proteins and reconstruct phylogenies of these components across the eukaryotes. We show that the evolutionary origins of multiple homologues of translation initiation factors in kinetoplastids, provide insights into the complex regulation of gene expression in trypanosomes. We also identify the points at which specific subunits associated with mRNA decapping and deadenylylation have been lost, and gene families associated with mRNA 5’ to 3’ degradation have expanded in individual eukaryotic lineages such as kinetoplastids. Finally, we demonstrate that many of the changes associated with eukaryotic mRNA metabolism are linked to changes in complexity in genome structure, particularly intron density, rather than changes in lifestyle such as parasitism. Overall, this analysis provides insights into the complexity of the gene expression pathways associated with LECA, and the factors underpinning subsequent changes to this machinery across the full diversity of the eukaryotes.

## Results

### Reconstruction of mRNA metabolism evolutionary pathways

We produced a set of 1655 non-redundant eukaryotic protein sequences involved in mRNA metabolism from the *Saccharomyces cerevisiae*, Ensembl and other genome databases ([20, 35]; Table A in S1 Table) and transcriptome datasets from the MMETSP transcriptome reference database [46, 47]. A reverse BLAST search of all the sequences against the NCBI nr-database was performed and sequences assessed for domains from the PFAM database [48], to confirm they were true homologues of proteins involved in mRNA maturation, translation initiation and mRNA degradation before using Bayesian and Likelihood phylogenetic methods to reconstruct their evolutionary ancestry (S2–S6 Tables).

### The exon junction complex is conserved in eukaryotes and shows a positive correlation to intron density

**Fig 1:**
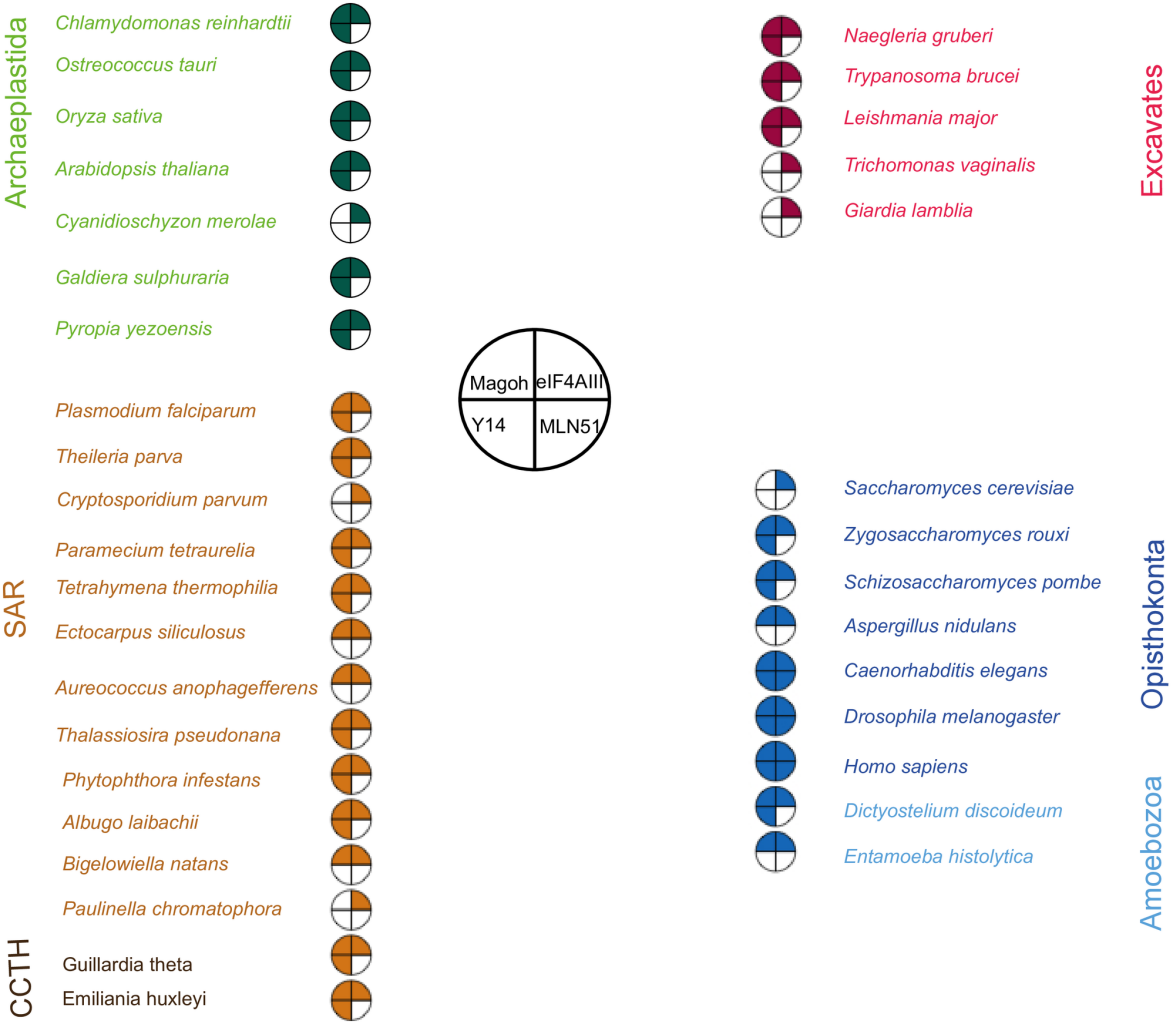
Coulson plot showing the presence/absence of exon junction complex components eIF4AIII, Magoh, Y14 and MLN51 in selected species representative of the eukaryotic diversity. The coloured segments of the plot indicates that the protein is present and the numeral denotes the number of homologues identified. The blank segments denote that homologues of these proteins were not identified in the corresponding species.

Homologues to the core components of the Exon Junction Complex (EJC); eIF4AIII, Magoh and Y14 were identified from genome sequences of 58 species including members of the six super-groups of eukaryotes (Archaeplastida, Amoebozoa, Opisthokonta, SAR, CCTH, and Excavata; Tables A-D in S2 Table). eIF4AIII, Magoh and Y14 were found to be present in all major eukaryotic super-groups but Magoh and Y14 are selectively absent in organisms with low intron densities (introns per gene) such as *Saccharomyces cerevisiae*; 0.003 introns per gene [25] and Cyanidioschyzon *merolae*; 0.005 introns per gene [49] (Fig 1). Another EJC component, MLN51, was shown to have evolved within the Holozoa, after the differentiation from fungi. Whilst MLN51 was not identified in fungi, it was identified in all other sub-groups within the Opisthokonta; such as in Choanomonada (*Monosiga brevicollis*) and in Metazoa (Table D in S2 Table).

**Fig 2:**
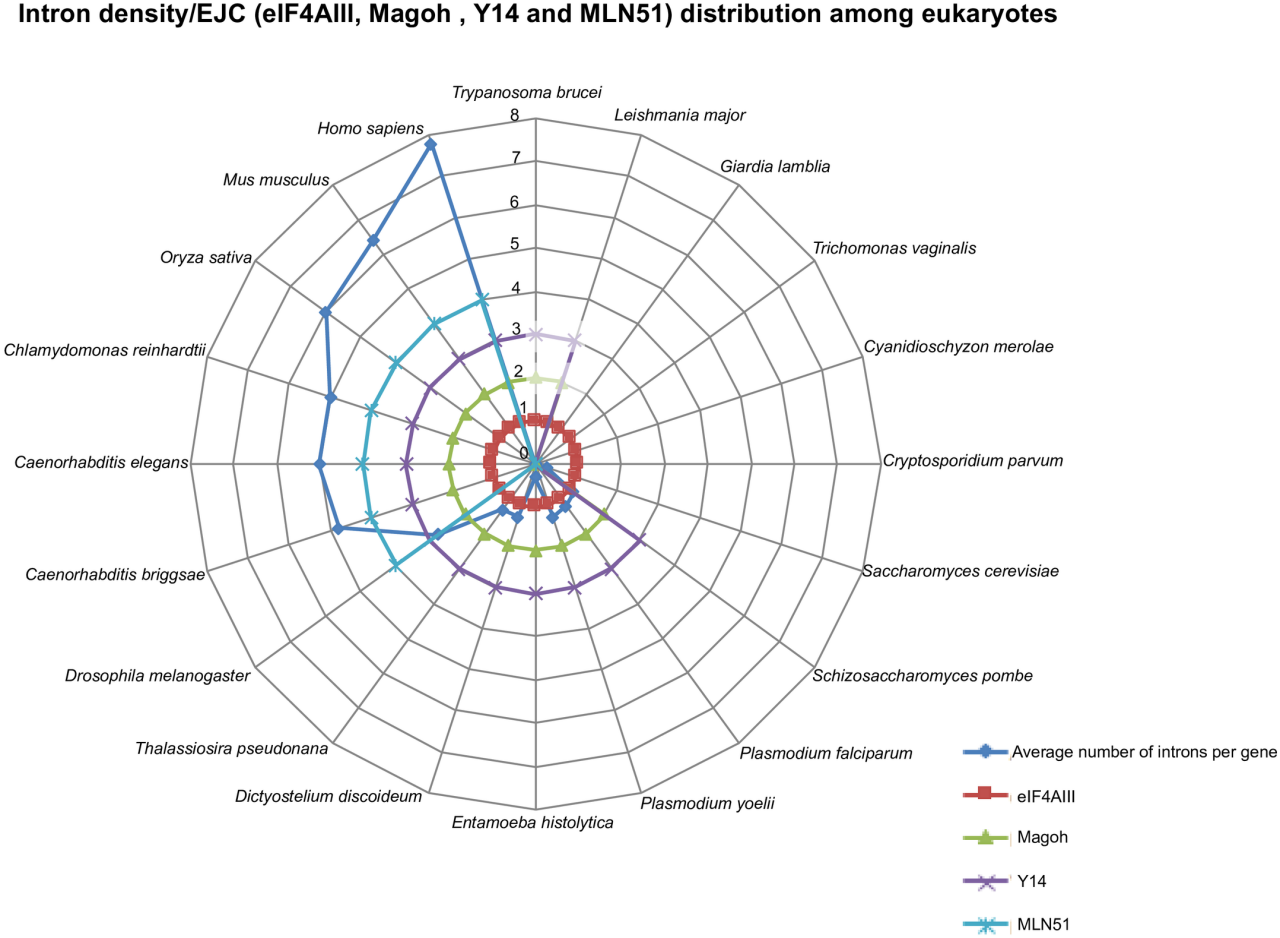
Intron density/EJC (eIF4AIII, Magoh, Y14 and MLN51) distribution among eukaryotes. The intron density (here: average number of introns per gene) is plotted for different eukaryotes. Adapted from Roy and Gilbert (2006). From top right: *Trypanosoma brucei*, *Leishmania major*, *Giardia lamblia*, *Trichomonas vaginalis*, *Cyanidioschyzon merolae*, *Cryptosporidium parvum*, *Saccharomyces cerevisiae*, *Candida albicans*, *Paramecium aurelia*, *Schizosaccharomyces pombe*, *Plasmodium falciparum*, *Plasmodium yoelii*, *Entamoeba histolytica*, *Dictyostelium discoideum*, *Thalassiosira pseudonana*, *Drosophila melanogaster*, *Caenorhabditis briggsae*, *Caenorhabditis elegans*, *Chlamydomonas reinhardtii*, *Oryza sativa*, *Arabidopsis thaliana*, *Mus musculus*, *Homo sapiens*. The EJC proteins are colour coded to show presence/absence in the various species represented: eIF4AIII is conserved and present in all species Magoh and Y14 are present mostly in intron-rich species, very diverged (represented in paler colours) as seen in *Trypanosoma brucei* and *Leishmania major* or not present in intron-poor species MLN51 is present only in animals.

We searched for the factors that may have underpinned the expansion and loss of mRNA metabolism proteins in eukaryotes. Many parasitic eukaryotes still retain complete EJC components (*Plasmodium falciparum* and *Plasmodium yoelii*), while some free-living species such as *Cyanidioschyzon merolae* have lost Magoh and Y14 indicating that reduction of the EJC is not explicitly linked to the transition to a parasitic lifestyle (S2 Table). We noted an association between intron density and the number of EJC components retained, as illustrated in Fig 2. Typically, organisms with low intron density retain only minimal sets of EJC components, whereas more intron-rich relatives retain a complete EJC. Within the Excavata, neither Magoh nor Y14 were present in *Giardia lamblia* which retains one documented intron or in *Trichomonas vaginalis* which has no known introns [50, 51], but both subunits are present in the heterolobosean species, *Naegleria gruberi*, which has a moderate intron density of 0.7 per gene [18]. Similarly, the red alga *C*. *merolae* and the yeast *S*. *cerevisiae*, which have reduced EJC complements, have far lower intron densities, respectively 0.005, and 0.03 [25, 52] than other red algae with intron densities 0.7–2.5, [53, 54, 55] or Opisthokonta with intron densities 0.9–8.1, [56] included in the analysis (Fig 1).

A notable exception to this rule were parasitic members of the kinetoplastids (such as *Trypanosoma brucei* and *Leishmania major*). These organisms typically possess extremely few introns (~0.0002 per gene [3, 57] but retain both Magoh and Y14. The kinetoplastid Y14 is extremely divergent, with unique differences to all other eukaryotes, including the loss of six residues that are universally conserved in all other species (S1 Fig). As the kinetoplastids utilise *trans*-splicing for expression of the nuclear genome [58] it is possible that the retention and divergence of Y14 in the kinetoplastid population might be complimentary to an alternate role of a modified Magoh and or Y14 complex in *trans*-splicing. However, dinoflagellates, which also perform *trans*-splicing [59] possess a much more conventional Y14 isoform (Table B in S2 Table), hence this difference, if indeed the reason for the unusual situation observed in kinetoplastids, is likely to be lineage-specific.

### Multiple independent duplication events of translation initiation factors in the Excavata and SAR groups

**Fig 3:**
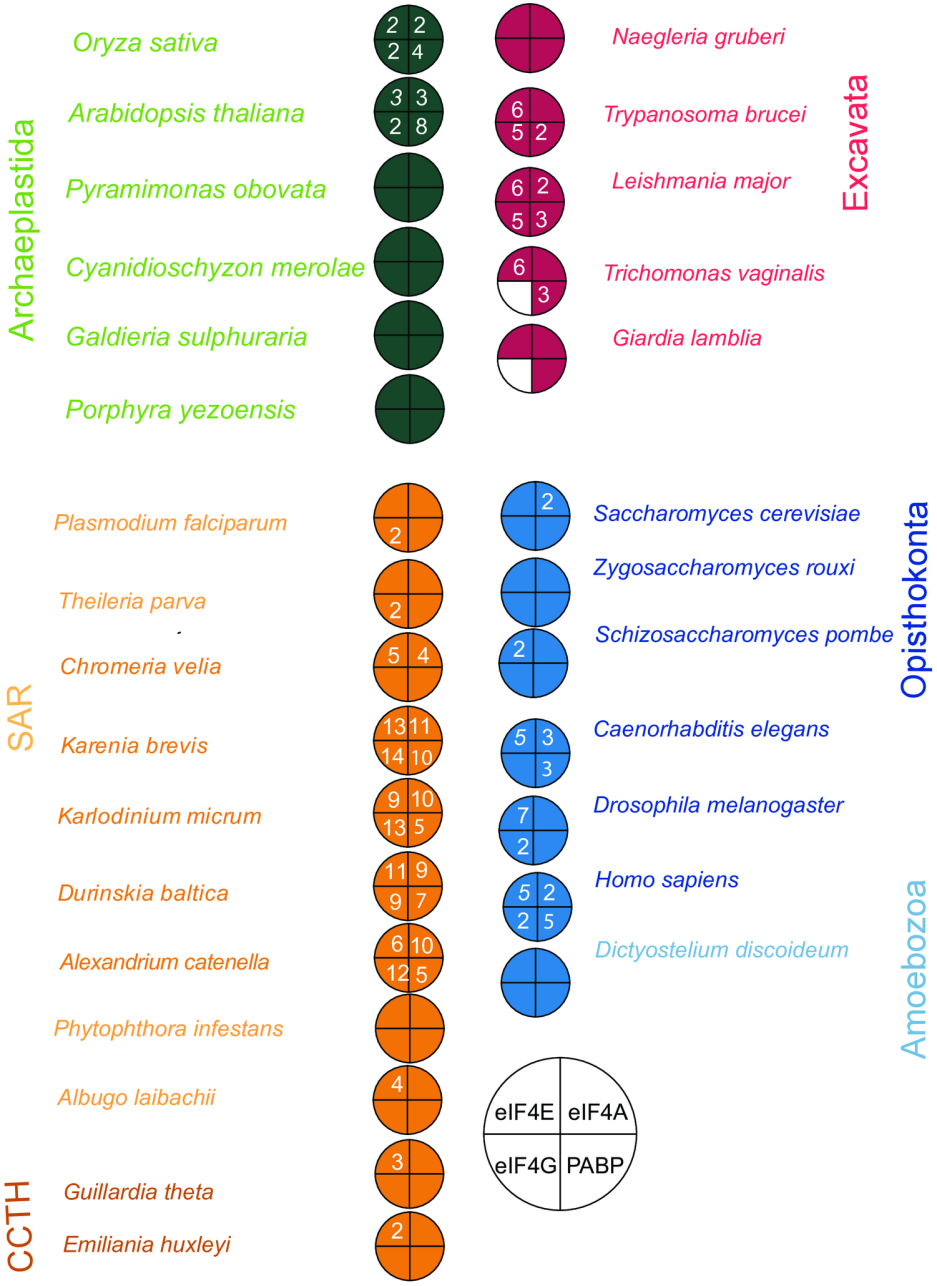
Coulson plot showing the presence/absence of eIF4A, eIF4E, eIF4G & PABP in selected species representative of the eukaryotic diversity. The coloured segments of the plot indicates that the protein is present and the numeral denotes the number of homologues identified. The blank segments denote that homologues of these proteins were not identified in the corresponding species.

Homologues of the translation initiation factors eIF4E and eiF4G, and the Poly(A) binding proteins were identified in other organisms of the Excavata and SAR groups (Tables A-D in S3 Table) as described in the methodology section. A graphical summary of the origin of each protein is provided in Fig 3, and illustrations of each key domain architecture, as inferred using PROSITE [60] from a selected range of species are shown in figures S2–S4 Figs. All the translation initiation factors were conserved across all the eukaryotic super-groups, hence were presumably present in the LECA. Consistent with previous studies [61] eIF4G was not detected in the *Metamonada* species, *Giardia* and *Trichomonas* of the Excavata group; suggesting that it is probably absent from or extremely divergent in these organisms.

**Fig 4:**
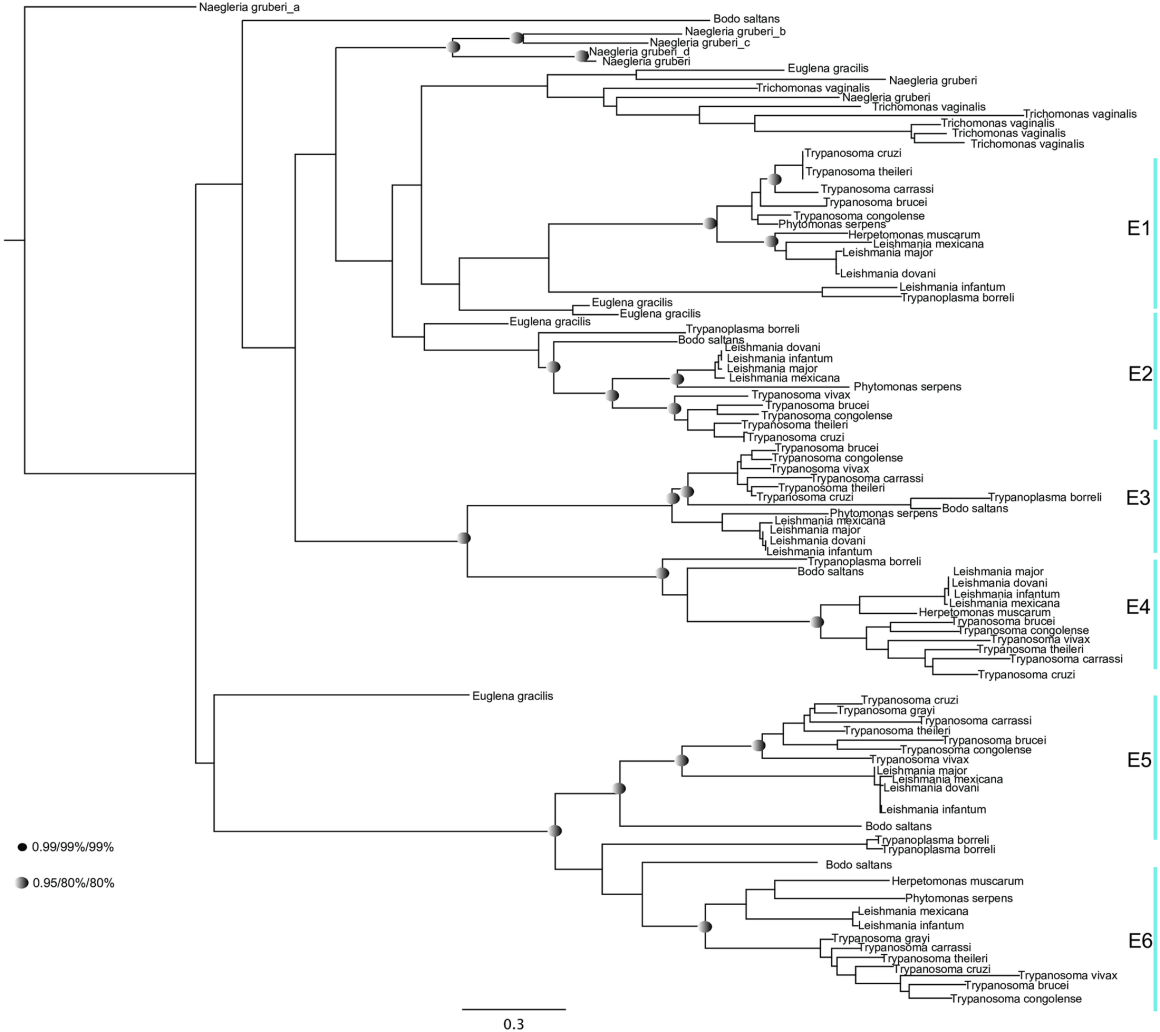
Phylogenetic analysis of eIF4E proteins in selected excavates using Mr Bayes (Posterior probability), PhyML & RaxML (Maximum Likelihood methods). The tree is shown in the Bayesian topology. Numerical values at the nodes of the tree (x/y/z) indicate statistical support by MrBayes, PhyML and RAxML, posterior probability, bootstrap and bootstrap, respectively. Values for highly supported nodes have been replaced by symbols as indicated. There are no values on branches that are not well supported.

**Fig 5:**
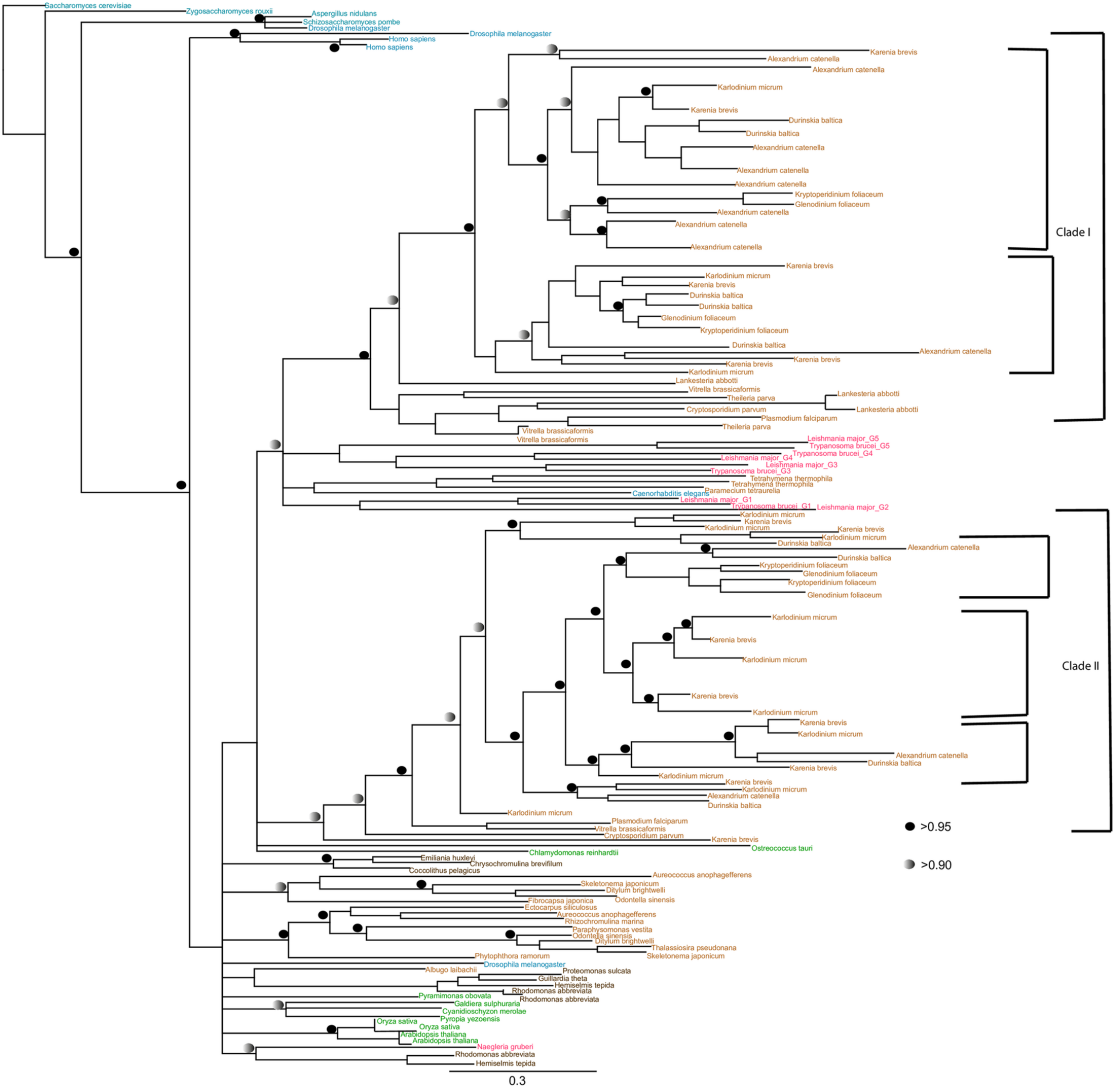
Phylogenetic analysis of eIF4G in selected eukaryotes using Mr. Bayes (Posterior probability). Values for highly supported nodes have been replaced by symbols as indicated. Numbers indicate posterior probability values for Mr Bayes.

**Fig 6:**
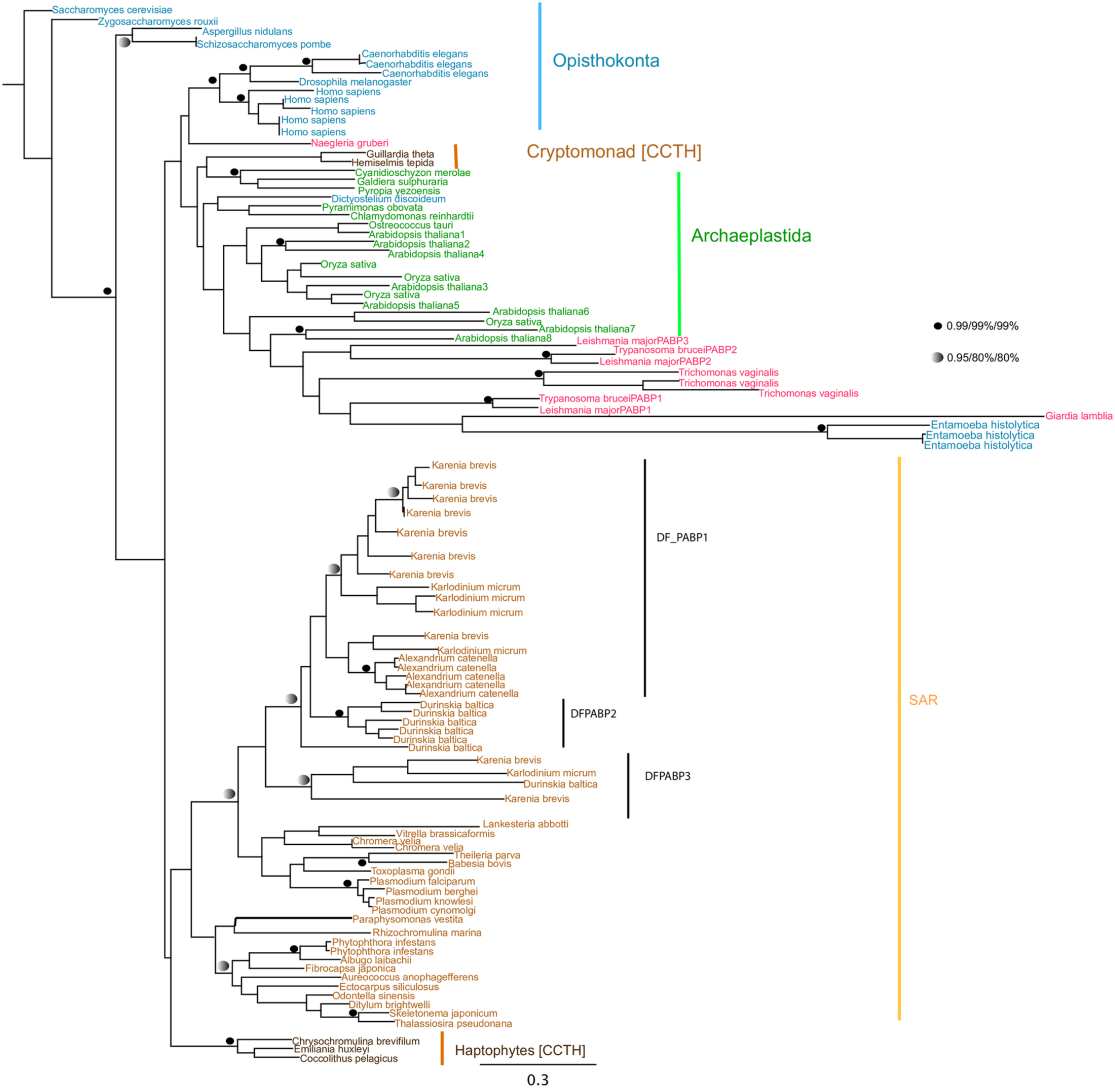
Phylogenetic analysis of PABP in selected eukaryotes using Mr Bayes (Posterior probability), PhyML & RaxML (Maximum Likelihood methods). Values for highly supported nodes have been replaced by symbols as indicated. Numbers indicate Bayesian posterior probabilities and bootstrap support for PhyML and RAxML respectively.

We additionally identified duplicated isoforms in multiple lineages of eIF4E (Fig 4), eIF4G (Fig 5), and PABP (Fig 6). Overviews of the duplication events inferred for each protein are provided in Tables A-D in S3 Table. For eIF4E, we identified several expansions in members of the Excavata, including pathogenic kinetoplastids (Fig 4). None of these homologues belong to the metazoan Class I, II and III eIF4E proteins [62]. Several of the eIF4E orthologues have divergent domain architectures: for example, *T*. *brucei* eIF4E3 and eIF4E4 possess a characteristic N-terminal extension, whereas *T*. *brucei* eIF4E6 and *Giardia*4E_a lack a recognisable eIF4E domain (S2 Fig). Phylogenetic analysis revealed that orthologues of five of the six eIF4Es present in *T*. *brucei* was also present in the free-living *Bodo saltans*, while the remaining subunit, eIF4E1, appears to be orthologous to two proteins encoded in the photosynthetic euglenid *Euglena gracilis* (Fig 4). Thus, duplications of the eIF4E family in trypanosomes precede the origins of parasitism in this lineage.

For eIF4G, we identified independent duplication events in the kinetoplastids and dinoflagellates (Fig 5). Trypanosome eIF4Gs (eIF4G1 to 5) resolve into two clades of eIF4G1/2/5, and of eIF4G3/4 [37] and orthologues were identified in bodonids, indicating that the expansion of the kinetoplastid eIF4G repertoire is not associated with the origin of parasitism (Fig 5). In contrast, we could not find expansions of eIF4G orthologues SAR group taxa other than dinoflagellates, such as chromerids and apicomplexans, pinpointing its origin to within this lineage (Fig 5). All the sequences studied contained a characteristic MIF4G domain (S3 Fig), although we noted an extra eIF4GI domain at the N-terminal of *Saccharomyces cerevisiae* and W2-eIF4G1-like domain at the C-terminal domain of *Homo sapiens*, suggesting that lineage-specific elaborations to domain architecture have occurred.

Finally, for PABP proteins, we identified three duplicated isoforms in trypanosomes, both in parasitic kinetoplastids and free-living bodonids, indicating that this duplication preceded the origins of parasitism in this lineage (Fig 6). We additionally identified independent expansions in PABPs in dinoflagellates and in land plants, that were not found in other SAR group or archaeplastid taxa (Fig 6). We found limited structural differences between PABP sequences between different lineages, with a representative set of sequences containing the 4 RRM domains and 1 PABP domain at the C-terminus characteristic of all PABP proteins (S4 Fig).

### Expansions and reductions within mRNA turnover proteins in eukaryotes

We investigated the evolutionary distribution of proteins involved in mRNA degradation within our dataset (Tables A-K in S4 Table). These included the deadenylase protein sequences; Ccr4/Caf1/Not, Caf40 and Pan2/Pan3, the 5’ to 3’ exonucleases Xrn1 and Rat1p; and the 5’ decapping enhancers Dhh1, Dcp1, PAT1 and SCD6. All the subunits of the mRNA deadenylylation, decapping and the 5’ to 3’ mRNA degradation pathways were broadly conserved in all eukaryotic super-groups, and were inferred to be present in the LECA (S4–S6 Tables).

**Fig 7:**
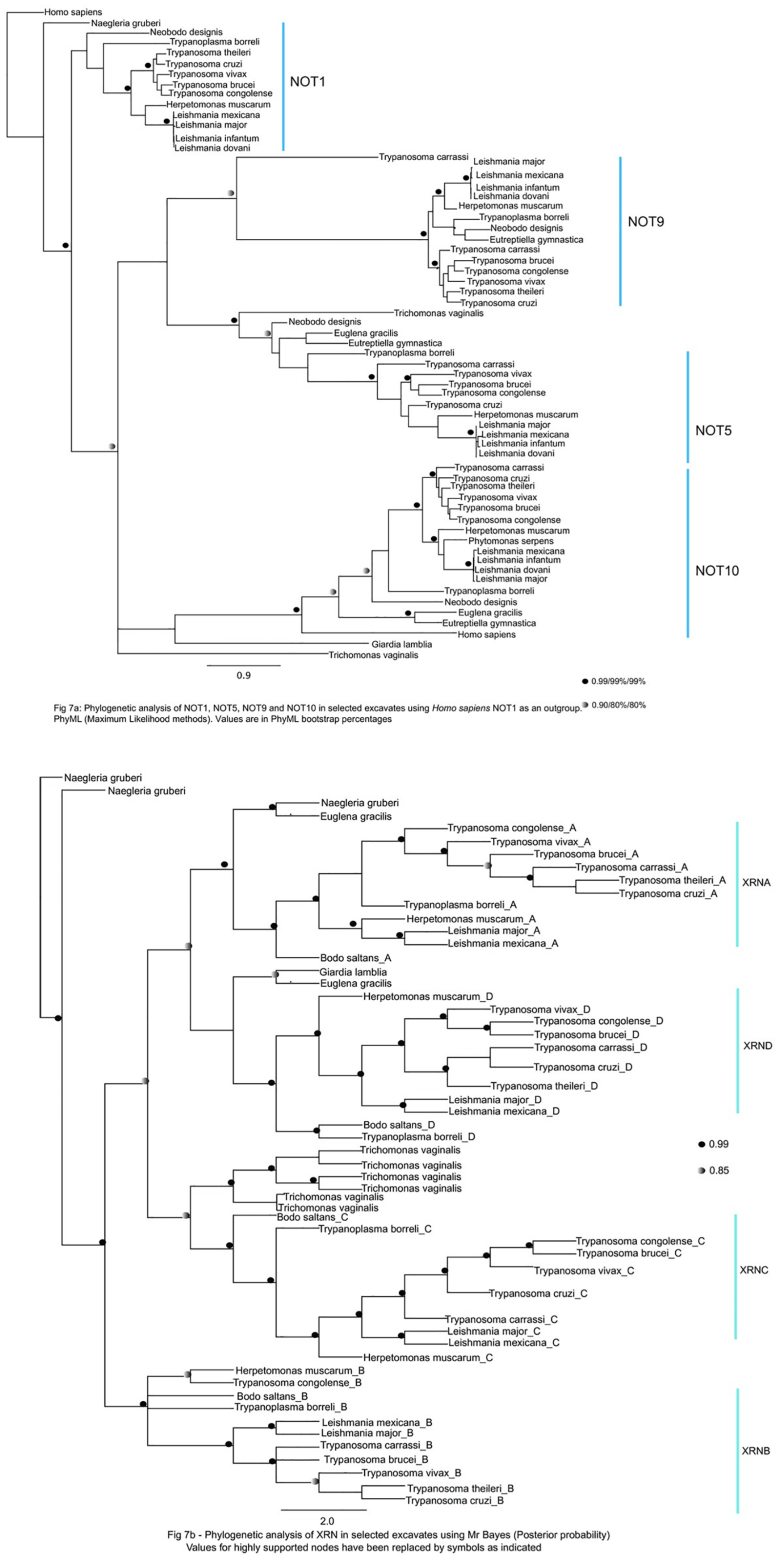
Phylogenetic analysis of mRNA degradation proteins in selected excavates. A) Phylogenetic analysis of NOT1, NOT5, NOT9 and NOT10 proteins using *Homo sapiens* NOT1 as an outgroup. Numbers indicate Bayesian posterior probabilities and bootstrap support for PhyML and RaxML respectively. B) Phylogenetic analysis of XRN proteins using Mr Bayes (Posterior probability). Values for highly supported nodes have been replaced by symbols as indicated.

We identified independent expansions in specific complexes in the ancestors of individual eukaryotic lineages. Two subunits of the modified Not complex (Caf40/Not9 and Not10) that were previously only known in *Trypanosoma brucei* were found [44]. in other parasitic kinetoplastids (Fig 7a in Fig 7), and in the free-living trypanosomatids, the bodonids. Thus, the origins of the expanded Not complex (and presumably the interactions between Not9, Not10, Not1 and Caf1 [44] are not associated with parasitism in kinetoplastids. Similarly, orthologues of the variant ribonucleases XRNB and XRNC, which were previously only known in parasitic kinetoplastids [45] were identified in the bodonids (Fig 7b in Fig 7).

We additionally identified independent losses of different components of the mRNA turnover machinery from individual eukaryotic lineages. In the most extreme case, the deadenylylation proteins, Ccr4, Caf40 as well as the decapping enhancers; PAT1 and SCD6 (known to bind eIF4G) are absent in *Giardia lamblia*, suggesting that only a minimal mRNA degradation machinery is present in this species. However, *Giardia* retains the 5’ decapping enhancer protein Dhh1 (Table A in S5 Table), as did all other species examined, indicating that this protein is presumably functionally indispensable for mRNA decay. We additionally identified independent losses of the decapping enhancer proteins Dcp1 in Kinetoplastea species; trypanosoma and bodonids, and Pat1 in certain members of the kinetoplastids and apicomplexans (Tables B and D in S5 Table). Thus, the reductions in the mRNA decay pathways are not specifically linked to the origins of pathogenicity.

## Discussion

**Fig 8:**
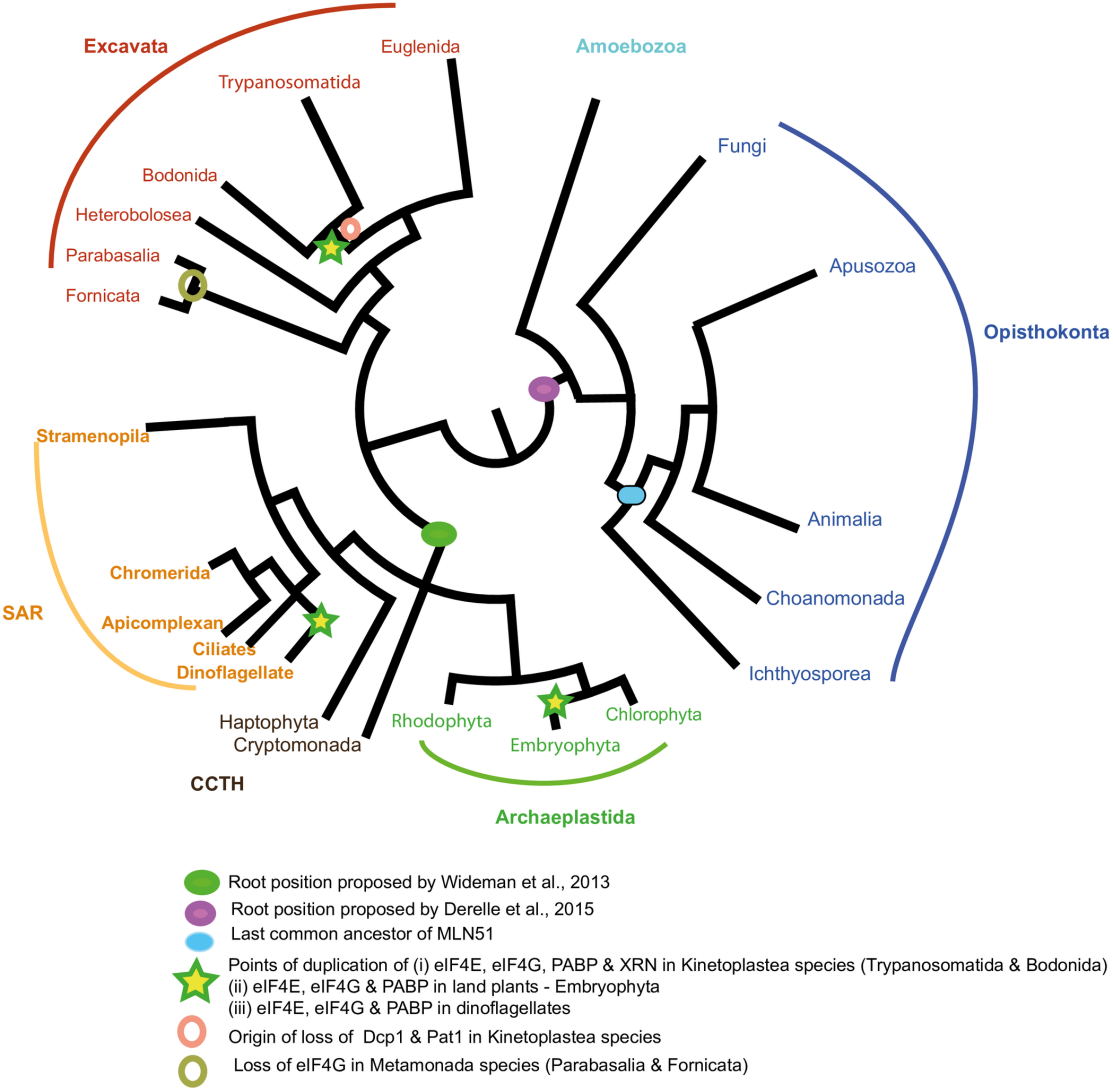
A schematic diagram of the origins, secondary loss, and expansions of key subunits discussed in this study, plotted onto a schematic tree of the eukaryotes [15, 22, 10]. The most probable position of each event was identified using Mesquite [80]. A full tree, containing all of the species used for analysis in this study, is shown in figure S6 Fig. The following proteins analysed in this study were found to be present in the LECA using parsimony analysis on Mesquite from both root positions [22, 10]: EJC proteins (eIF4AIII, Magoh, Y14); Translation Initiation factors (eIF4AI, eIF4E, eIF4G); PABP & mRNA degradation proteins analysed in this study. The exception is the EJC protein, MLN51 which was found to be present in Opisthokonta to the exclusion of Fungi.

In this study, we have elaborated on the origins and diversification of eukaryotic mRNA processing pathways, using published genome and transcriptome datasets. With the availability of the Marine Microeukaryote Transcriptome Sequencing Project (MMETSP) datasets [46] we have incorporated close relatives of major parasitic eukaryote lineages (for example, the free-living bodonid relatives of kinetoplastid parasites, and dinoflagellate and chromerid relatives of parasitic apicomplexans). This enables us to gain unprecedented insights into some unusual and hitherto underexplored lineages (Excavata, SAR, CCTH), and investigate the evolutionary transitions that have occurred in parasitic eukaryotes in comparison to their free-living relatives. We present a schematic overview of these events in Fig 8.

Almost all the proteins involved in translation initiation and mRNA degradation investigated in this study are conserved in all lineages, hence were presumably present in the LECA. This was verified using parsimony analysis on Mesquite from multiple root positions [6, 10]. The only exception was the EJC component, MLN51, which is restricted to the Opisthokonta group (Fig 8). The complexity observed in the mRNA metabolism pathways present in ancient eukaryotes mirrors a growing body of evidence for a complex cellular, nuclear and genome organisation in the LECA [63, 64, 65, 66 67, 68, 69]. Single gene trees of each factor broadly recover the 6-established eukaryotic super-groups, indicating a probable vertical inheritance of each complex (Figs 5, 6 and 8). It will be interesting to compare the conserved proteins involved in mRNA metabolism to proteins present in probable close relatives and contributors to the eukaryotic lineage, such as Lokiarchea [70] to determine how much of the mRNA metabolism pathways evolved *de novo* in eukaryotes. It is already known, for example, that the Poly(A)-binding protein (PABP) is probably generated from ancestral RRM precursors from prokaryotes and XRN1 and Rat1 are 5’ to 3’ exoribonucleases, with unique domains, which were likely generated at the beginning of eukaryotic evolution [64]. Similar comparisons of conserved eukaryotic and non-eukaryotic protein datasets may provide valuable insights into the processes underpinning the origins of eukaryotic cells.

Since their radiation, independent duplication events have occurred within translation initiation and mRNA turnover subunits in different eukaryotic lineages. For example, we identify multiple eIF4E, eIF4G and PABP paralogues in dinoflagellates and kinetoplastids, indicating independent gene duplication events in each lineage (Figs 4, 5, 6 and 8). These observations fit with a growing body of evidence for convergent evolution between kinetoplastids and alveolates, which may reflect the extremely fast sequence evolution and extensive paralogy observed in these lineages [71]. It remains to be determined what biological roles the different paralogous copies of mRNA metabolism proteins perform, particularly for dinoflagellates. For these, experimental methods such as biochemical assay or analysis of expression trends, both of which are feasible and of expanding importance for dinoflagellate biology [72, 73], may be useful in inferring function.

We have additionally found multiple species which possess diminished versions of the ancestral eukaryotic mRNA metabolism machinery (Fig 8). The most extreme case of this was in *Giardia lamblia* which has lost multiple subunits associated with the EJC (Magoh and Y14), translation initiation machinery (eIF4G) and mRNA degradation machinery (Ccr4, Caf40, Pat1, Scd6) (Figs 1 and 3; S4 and S5 Tables), consistent with the extremely reduced number mRNA turn-over proteins observed in this species [41, 74] Some of the duplication and reduction events identified in our data might have occurred concertedly. For example, the two eIF4E homologues present in *Giardia lamblia* are very distinct from homologues in other eukaryotes (S5 Fig), lacking consensus sites for binding eIF4G [61]. Given that eIF4G is absent from *G*. *lamblia* (Fig 3), it is possible that the divergent evolution of eIF4E occurred alongside the loss of conventional eIF4G from early diplomonads. It remains to be determined in this case whether *Giardia* can facilitate interactions between the mRNA 5’ cap and 3’ poly(A) tail via an alternative mechanism.

The taxonomic distribution of duplication and reduction events of different mRNA metabolism subunits allows for preliminary insights into their broader evolutionary consequences. For example, we note a correlation between changes in intron density and in the number of genes encoding EJC components (Fig 2). Previous studies have identified simplified EJC machineries in individual lineages, for example trypanosomes [75] but previously it has not been shown to be true across multiple eukaryotic groups. The lineage-specific reduction in EJC subunits in intron-poor species mirrors the situation observed for other factors involved in intron processing. For example, the intron-poor red alga *Cyanidioschyzon merolae* [76] not only lacks the Magoh and Y14 subunits of the EJC, but also apparently lacks the U1 (A & C) and U4/U6 subunits of the spliceosome, even though these subunits are otherwise broadly conserved across the eukaryotes, and the U1 snRNP is known in the closely related and intron-rich red alga *Galdieria sulphuraria* [77]. *Cyanidioschyzon merolae* might have therefore evolved a very unusual splicing pathway, dependent on an extremely reduced machinery. It remains to be determined whether the reduction in intron density precedes, or occurs concurrently to the reduction of EJC subunits in eukaryotes.

We additionally demonstrate that duplications of genes encoding proteins involved in translation initiation (eIF4E, eIF4G and PABP) and mRNA degradation (XRN), and the secondary loss of genes encoding for mRNA decapping enzymes (Dcp1 and Pat1) occurred prior to the divergence of pathogenic kinetoplastids from free-living relatives within the Euglenozoa such as bodonids and euglenids, indicating that they are not explicitly linked to the origins of parasitism in this lineage (Tables B-D in S5 Table; Fig 8). This progressive modification to nuclear mRNA metabolism in free-living relatives of the Euglenozoa reflects the situation observed for other RNA metabolism pathways in this lineage (e.g. mitochondrial RNA editing), but contrasts with mRNA metabolism in other parasitic eukaryotes (e.g. apicomplexan plastid RNA processing), in which specific changes to gene expression pathways may delineate the divergence of parasitic species from their free living relatives [78, 79]. Identifying the exact timing of these events, and their possible physiological consequences, will be facilitated by studying genomic sequences of both Diplonemea and Symbiontida organisms, which are respectively free-living and commensal sister groups to Euglenida and Kinetoplastea [79, 80].

In summary, this study illustrates that complex mRNA metabolism pathways were present in the LECA and demonstrates the gene gains and losses that have occurred in the evolution of mRNA in parasitic and free-living members of the Excavata and SAR groups thus showing innovations in these organisms that contribute to their unique differences in gene expression compared to other eukaryotes.

## Methods

To identify homologues of specific mRNA metabolism pathways across the eukaryotes, a selected set of query protein sequences involved in translation initiation and mRNA degradation from the *Saccharomyces cerevisiae* genome were retrieved from the *Saccharomyces* and the Ensembl genome databases ([81, 82]; Table B in S1 Table). Where the *Saccharomyces cerevisiae* genome lacked homologues, *Schizosaccharomyces pombe* polypeptides were used as initial query sequences. Finally, the protein sequences of specific subunits only identified in parasitic kinetoplastids and apicomplexans were added to the dataset. For example, for mRNA 5’ decay, the deadenylase protein sequences; Ccr4/Caf1/Not, Caf40 and Pan2/Pan3 from *Saccharomyces cerevisiae* and/or *Schizosaccharomyces pombe*, were supplemented with experimentally characterised sequences Not2, Not3, Not5, Not9 and Not10 sequences from *T*. *brucei*. A complete list of query protein sequences is provided in Tables A-I in S7 Table.

Homologues of the query sequences were searched for in various other genome and transcriptome databases, using for Blastp, PSI-BLAST and Tblastn searches using the Blosum 62 matrix [83] with a manual cut-off 1.00E10^-5^ in all instances (Table B in S1 Table). Representative organisms from the major eukaryotic super-groups whose genome sequences were either complete or near completion with a permanent draft available were selected with priority (Table B in S1 Table). Where no such sequences were available (for example, within dinoflagellates, and bodonids), combined transcriptome datasets were retrieved from a previously modified version of the MMETSP database that had previously been cleaned of potential contaminant sequences [46, 47, 84]. Other transcriptome datasets, which include, *Trypanoplasma borreli*, *Trypanosoma theileri*, *Trypanosoma carassii*, and *Euglena gracilis* were provided from the laboratories of Mark Carrington, Cambridge and Steve Kelly, Oxford [85, 86].

The identified homologue sequences were then parsed through the Pfam database [48] PROSITE [52] and the NCBI Conserved domain database [87] using default parameters and analysed for the presence of domains. Where these initial searches failed to identify candidate homologues, query sequences of closely related taxa were then used to search for homologues. For more divergent proteins, a Hidden Markov Model (HMM) [88] was used to identify homologues using an alignment of selected proteins from each family. All homologues that passed this second round of validation were uploaded in Geneious 7.1.8 [89] for further alignment and phylogenetic analysis.

Multiple sequence alignments were constructed with MUSCLE [28] using the Blosum 62 matrix and the following parameters: number of iterations—8, Gap extension penalty of 0.20, a Gap Open score of -1 and with a FASTA sequence output. The output alignments produced from MUSCLE were visually inspected and edited by hand using Geneious 7.1.8 [89] to remove gaps and the non-aligned regions at the N or C termini. Positions with a consensus (plurality) of gapped identities were removed, as were all positions upstream of the first residue with 70% and downstream of the last reside with 70% conservation; the sequences of each trimmed alignment are catalogued in Table A-I in S7 Table and the percentage of pairwise identity, identical sites, number of residues are listed in Tables A-O in S8 Table.

The model of evolution for each dataset was determined by Prot-Test [90] and the edited alignments of various protein sets of eukaryotes analysed were then used to construct phylogenetic trees to determine orthologues and paralogues using the MrBayes, PhyML and RAxML programmes in-built in Geneious [89] Bayesian trees were inferred using either of the three substitution models (GTR, Jones, and WAG), as determined by Prot-Test [90]1,100, 000 chains were run to check for convergence and a 100,000% burn-in was discarded. PhyML and RAxML trees were also inferred using substitution models determined by Prot-Test [90] Bootstrapping was performed for each PhyML and RaxML tree for 1000 replicates and the best tree topologies were inferred. The data analysed on this study (including all the alignments and phylogenetic trees) are now fully available as supplementary figures.

Coulson plots were used to prepare models illustrating the loss and gain of pathways in eukaryotes analysed in the study [91]. The coloured part of the plot means presence of the protein, with a number to denote whether more than one homologue was identified. The blank segments denote that homologues of these proteins were not identified in the corresponding species. Mesquite [92] was used to prove presence / absence of specific subunits and domain elaborations in the ancestors of extant taxa via a parsimony analysis. A schematic tree diagram containing all the species used for this, based published tree topologies [8, 93, 94, 95, 96, 97], which was used to describe analysed proteins as "conserved" or "non-conserved" is shown in Supplementary S6 Fig. Due to the ongoing uncertainty concerning the exact rooting position of the eukaryotes, two alternative positions, taken from [6; 10] were used to infer presence/ absence in the LECA.

## Supporting information

S1 Fig Y14 alignment in PDB structure of the exon junction complex, *H*. *sapiens*, 2JOS. (TIF)

S2 Fig eIF4E protein domains in various eukaryotes showing identified domains. Cartoon structures were created with PROSITE domain image creator and co-ordinates retrieved from NCBI Conserved domain search.
(TIF)

S3 Fig eIF4G protein domains in various eukaryotes showing identified domains. Cartoon structures were created with PROSITE domain image creator and co-ordinates retrieved from NCBI Conserved domain search.
(TIF)

S4 Fig PABP protein domains in various eukaryotes showing identified domains. Cartoon structures were created with PROSITE domain image creator and co-ordinates retrieved from NCBI Conserved domain search.
(TIF)

S5 Fig Multiple sequence alignment of eIF4E sequences *Homo sapiens (*Hsa*)*, *Giardia lamblia and Trichomonas vaginalis*; conserved tryptophan residues (W) are and shaded in yellow (*H*. *sapiens* & *G*. *lamblia*) and blue (*T*. *vaginalis*). (TIF)

S6 Fig A full tree, containing all of the species used for analysis in this study. (TIF)

S1 File This is a zipped file containing all the alignment data used in this study. (ZIP)

S1 Table Table A in S1 Table—List of proteins analysed on this study with corresponding names in the *Saccharomyces cerevisiae* and *Schizosaccharomyces pombe* organisms. Table B in S1 Table—Templates used for BLAST searches.
(XLSX)

S2 Table Table A in S2 Table—List of Magoh homologues identified in various eukaryotes. Table B in S2 Table—List of Y14 homologues identified by Blastp with corresponding e-values. Table C in S2 Table—List of MLN51 homologues identified by Blastp with corresponding e-values. Table D in S2 Table—List of eIF4AIII homologues identified by Blastp with corresponding e-values.
(XLS)

S3 Table Table A in S3 Table—List of eIF4AI homologues identified by Blastp with corresponding e-values. Table B in S3 Table—List of eIF4E homologues identified by Blastp with corresponding e-values. Table C in S3 Table—List of eIF4G homologues identified by Blastp with corresponding e-values. Table D in S3 Table—List of PABP homologues identified by Blastp with corresponding e-values.
(XLSX)

S4 Table Table A in S4 Table—List of Ccr4 homologues identified by Blastp with corresponding e-values. Table B in S4 Table—List of Caf1 homologues identified by Blastp with corresponding e-values. Table C in S4 Table—List of Pan2 homologues identified by Blastp with corresponding e-values. Table D in S4 Table—List of Pan3 homologues identified by Blastp with corresponding e-values. Table E in S4 Table—List of Caf40 homologues identified by Blastp with corresponding e-values. Table F in S4 Table—List of Not1 homologues identified by Blastp with corresponding e-values. Table G in S4 Table—List of Not2 homologues identified by Blastp with corresponding e-values. Table H in S4 Table—List of Not3 homologues identified by Blastp with corresponding e-values. Table I in S4 Table -List of Not4 homologues identified by Blastp with corresponding e-values. Table J in S4 Table -List of Not5 homologues identified by Blastp with corresponding e-values. Table K in S4 Table—List of Not10 homologues identified by Blastp with corresponding e-values.
(XLS)

S5 Table Table A in S5 Table—List of Dhh1 homologues identified by Blastp with corresponding e-values. Table B in S5 Table—List of Dcp1 homologues identified by Blastp with corresponding e-values. Table C in S5 Table—List of Dcp2 homologues identified by Blastp with corresponding e-values. Table D in S5 Table—List of Pat1 homologues identified by Blastp with corresponding e-values. Table E in S5 Table—List of Scd6 homologues identified by Blastp with corresponding e-values.
(XLS)

S6 Table Table A in S6 Table—List of Xrn1 homologues identified by Blastp with corresponding e-values. Table B in S6 Table—List of Xrn2 homologues identified by Blastp with corresponding e-values.
(XLS)

S7 Table Table A in S7 Table—The sequences from the trimmed alignment of eIF4A proteins. Table B in S7 Table—The sequences from the trimmed alignment of eIF4E proteins. Table C in S7 Table—The sequences from the trimmed alignment of eIF4G proteins. Table D in S7 Table—The sequences from the trimmed alignment of PABP proteins. Table E in S7 Table—The sequences from the trimmed alignment of NOT1 proteins. Table F in S7 Table—The sequences from the trimmed alignment of NOT5 proteins. Table G in S7 Table—The sequences from the trimmed alignment of NOT9 proteins. Table H in S7 Table—The sequences from the trimmed alignment of NOT10 proteins. Table I in S7 Table—The sequences from the trimmed alignment of XRN proteins.
(XLSX)

S8 Table Table A in S8 Table—eIF4E percentage identity, Table B in S8 Table—eIF4E non-identical residues. Table C in S8 Table—eIF4E identical residues. Table D in S8 Table—eIF4G percentage identity. Table E in S8 Table—eIF4G non-identical residues. Table F in S8 Table—eIF4G identical residues. Table G in S8 Table—PABP percentage identity. Table H in S8 Table—PABP non-identical residues. Table I in S8 Table—PABP identical residues. Table J in S8 Table—Percentage identify of the NOT complex analysed in this study. Table K in S8 Table—non-identical residues in the NOT complex analysed in this study. Table L in S8 Table—identical residues in the NOT complex analysed in this study. Table M in S8 Table -Percentage identify of the exoribonucleases (XRN) analysed in this study. Table N in S8 Table—Non-identical residues of the exoribonucleases (XRN) analysed in this study. Table O in S8 Table—Identical residues of the exoribonucleases (XRN) analysed in this study.
(XLSX)
