# Supplementary figures and images for: Multispecies reconstructions uncover widespread conservation, and lineage-specific elaborations in eukaryotic mRNA metabolism
Source: PLoS One. 2018 Mar 21;13(3):e0192633. doi: 10.1371/journal.pone.0192633 (PMC5862402; doi:10.1371/journal.pone.0192633)

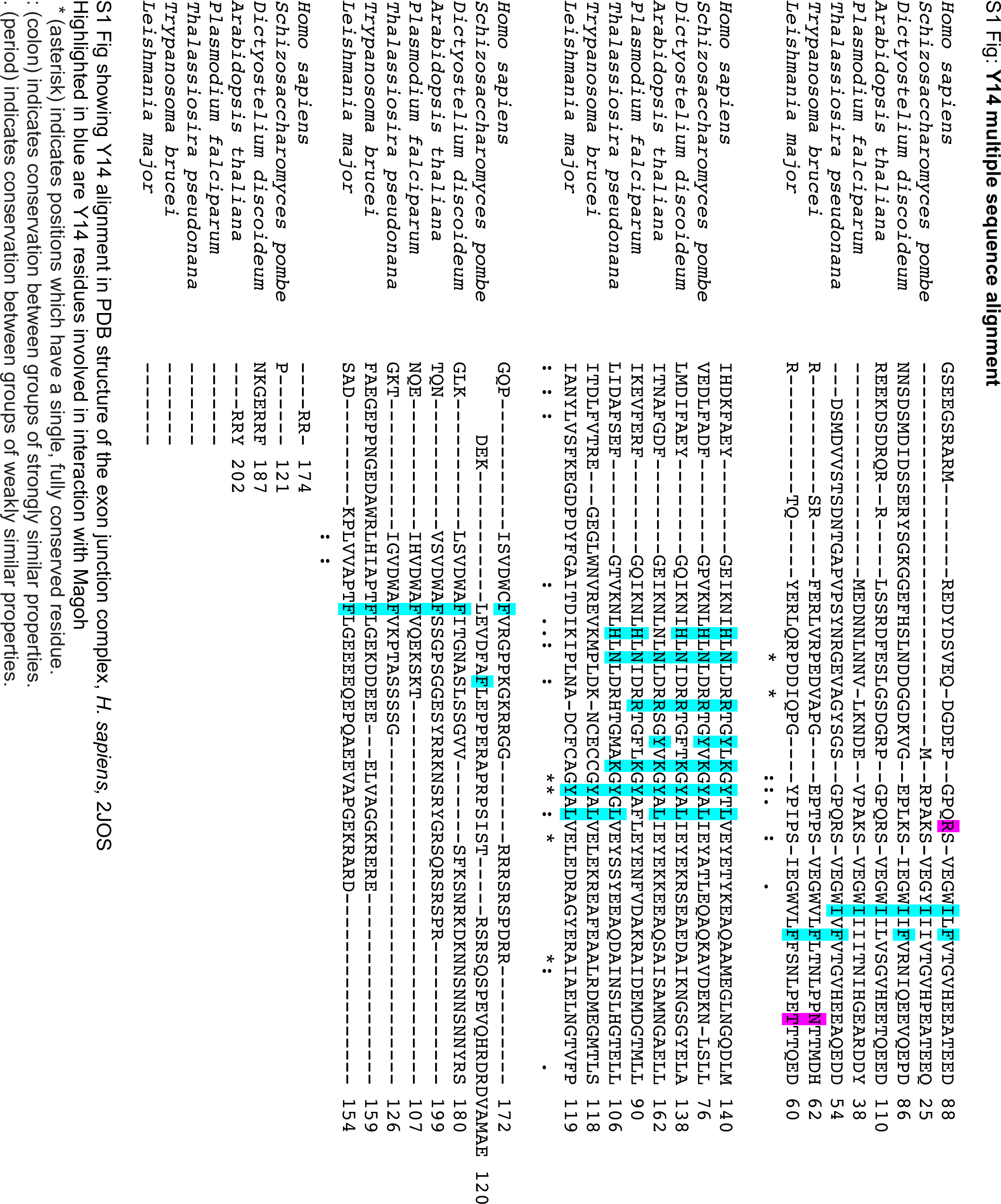

Supplement: S1 Fig — (TIF) [file pone.0192633.s001.tif]

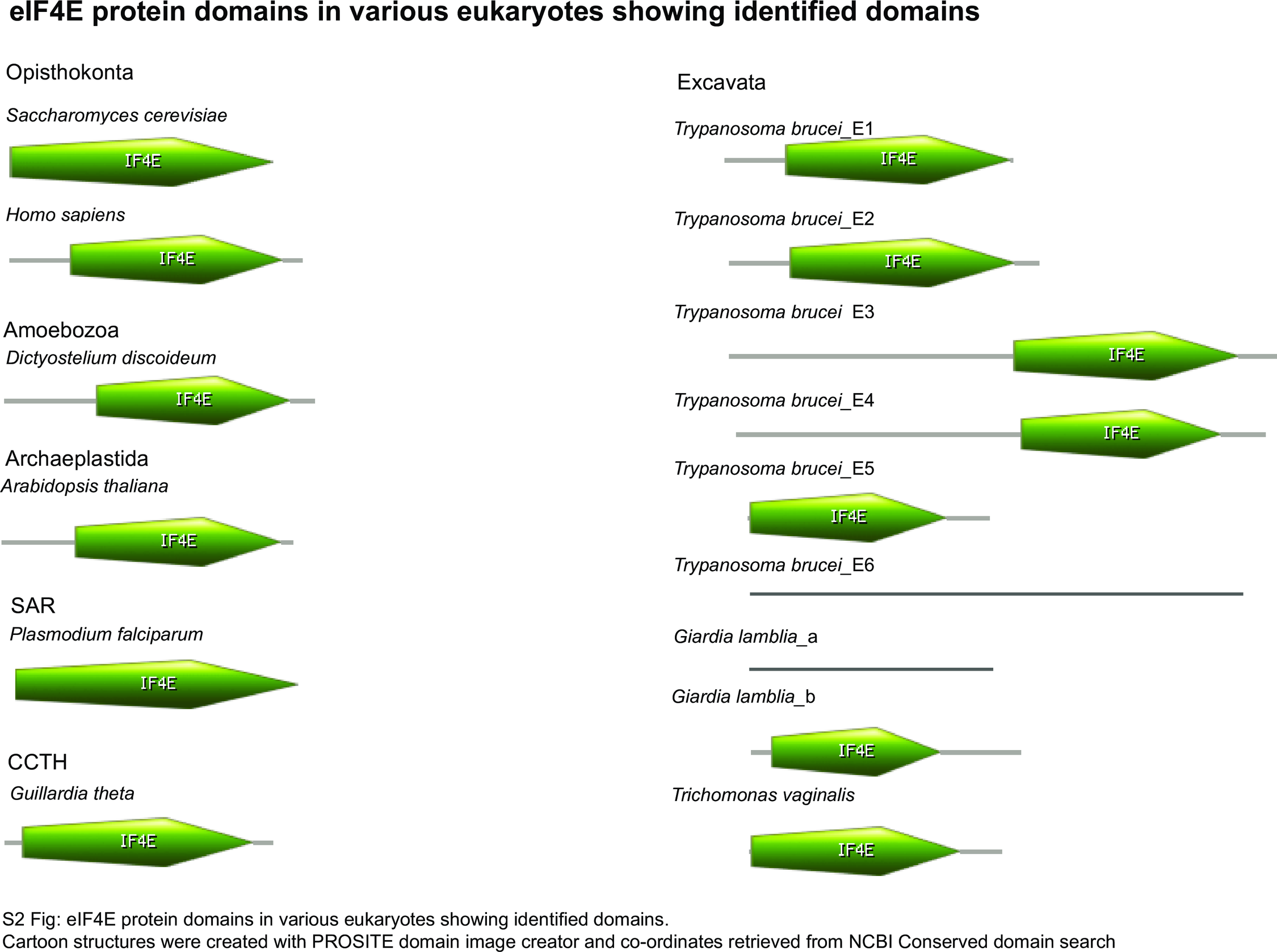

Supplement: S2 Fig — Cartoon structures were created with PROSITE domain image creator and co-ordinates retrieved from NCBI Conserved domain search. (TIF) [file pone.0192633.s002.tif]

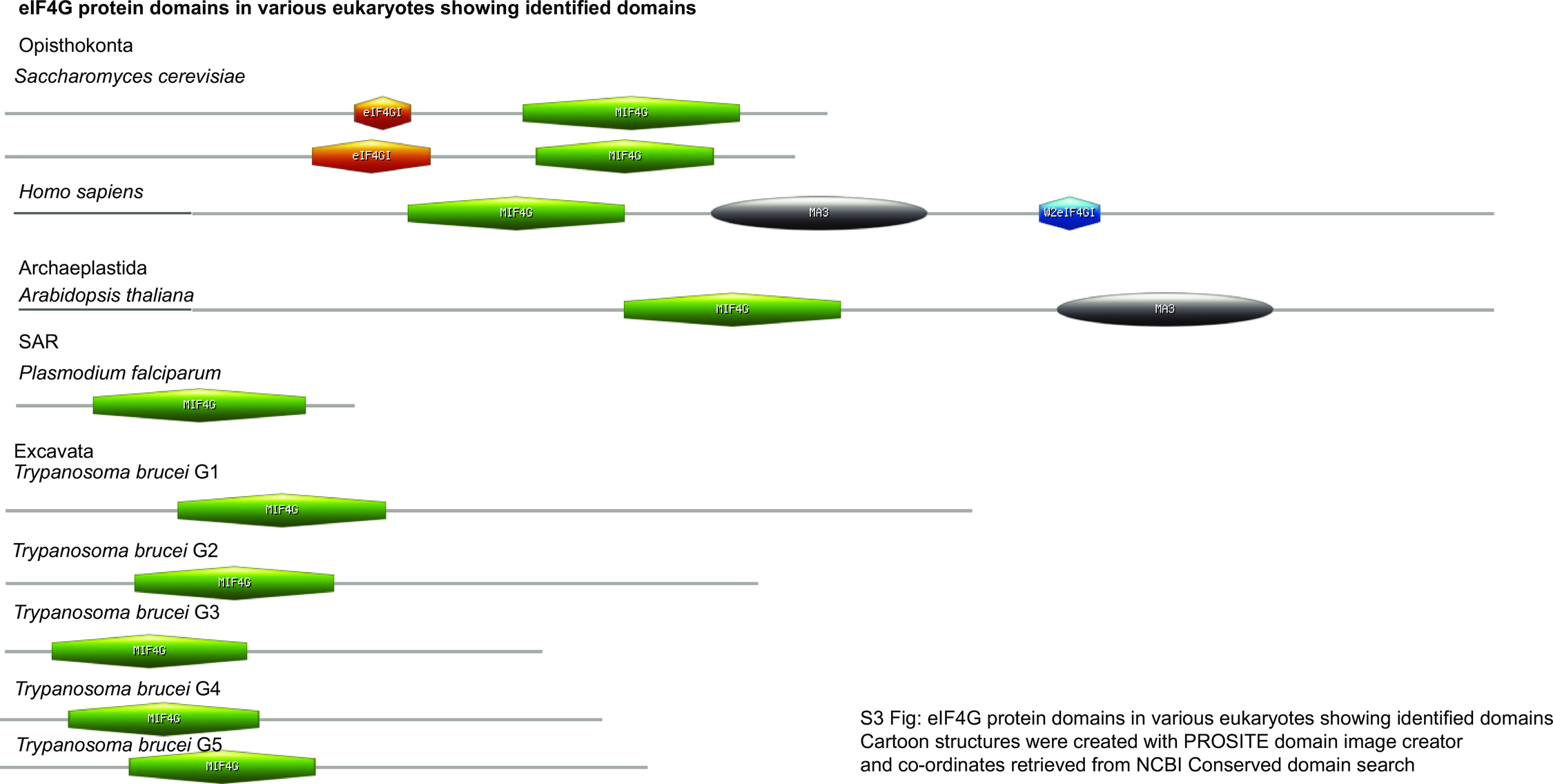

Supplement: S3 Fig — Cartoon structures were created with PROSITE domain image creator and co-ordinates retrieved from NCBI Conserved domain search. (TIF) [file pone.0192633.s003.tif]

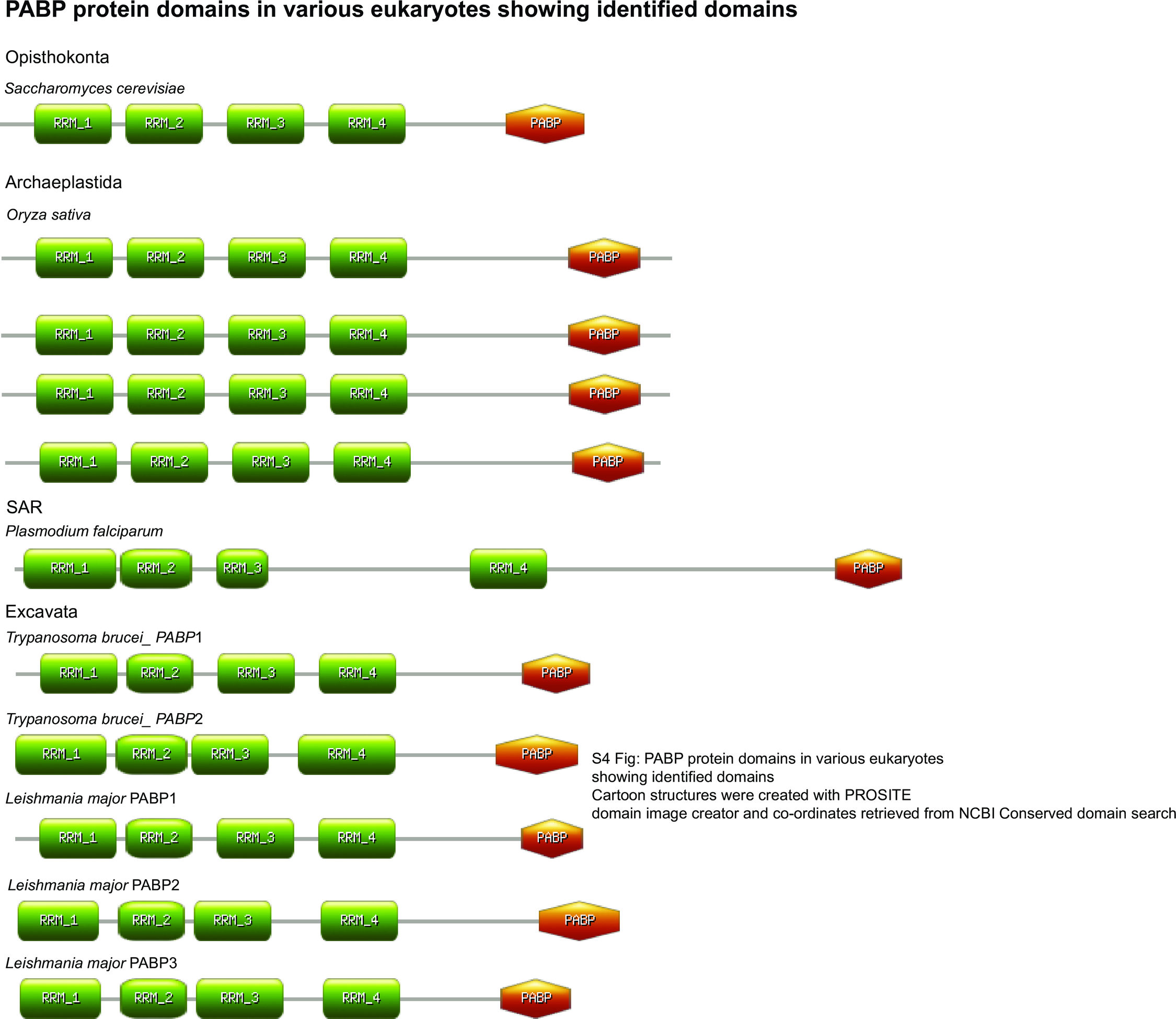

Supplement: S4 Fig — Cartoon structures were created with PROSITE domain image creator and co-ordinates retrieved from NCBI Conserved domain search. (TIF) [file pone.0192633.s004.tif]

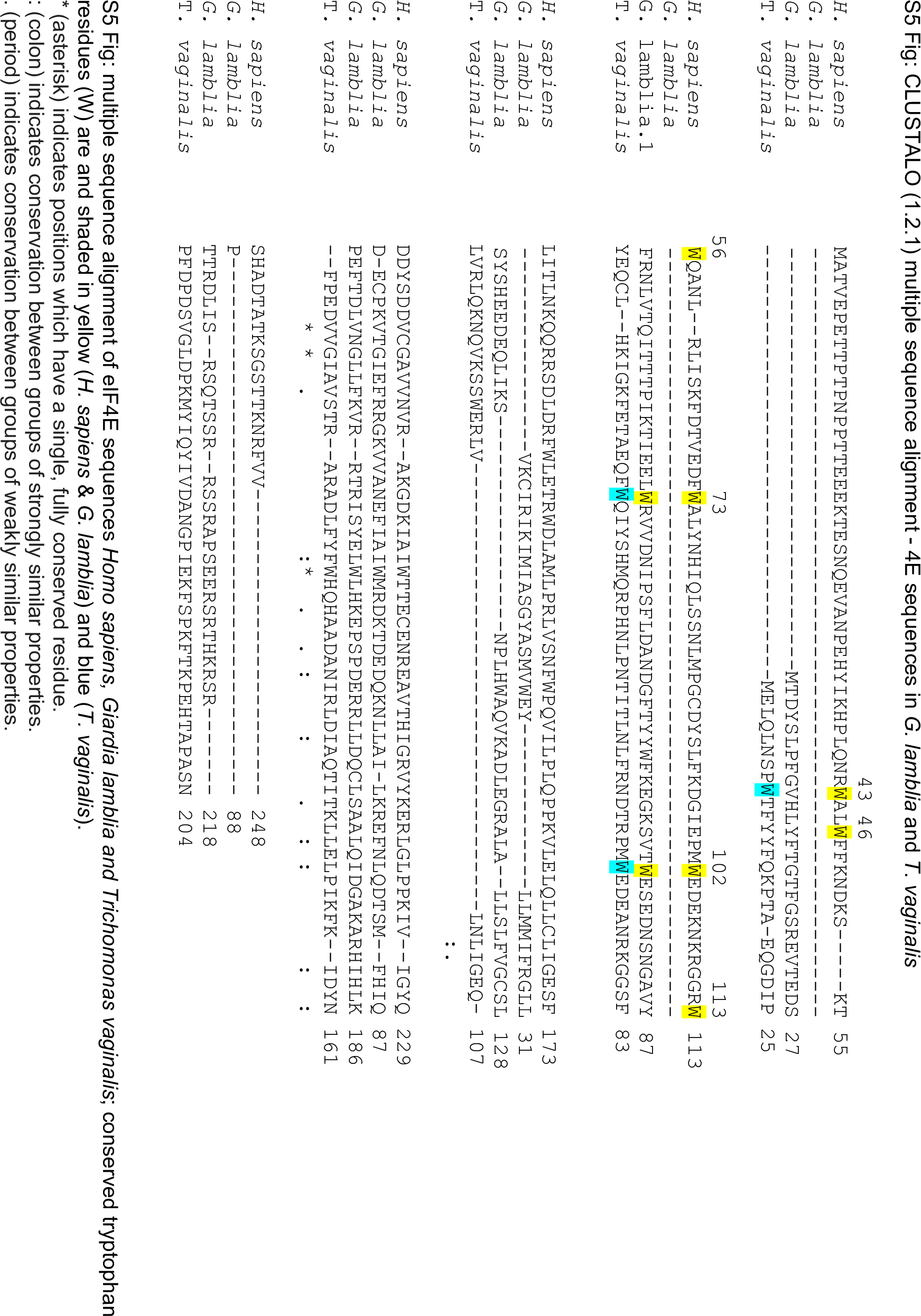

Supplement: S5 Fig — (TIF) [file pone.0192633.s005.tif]

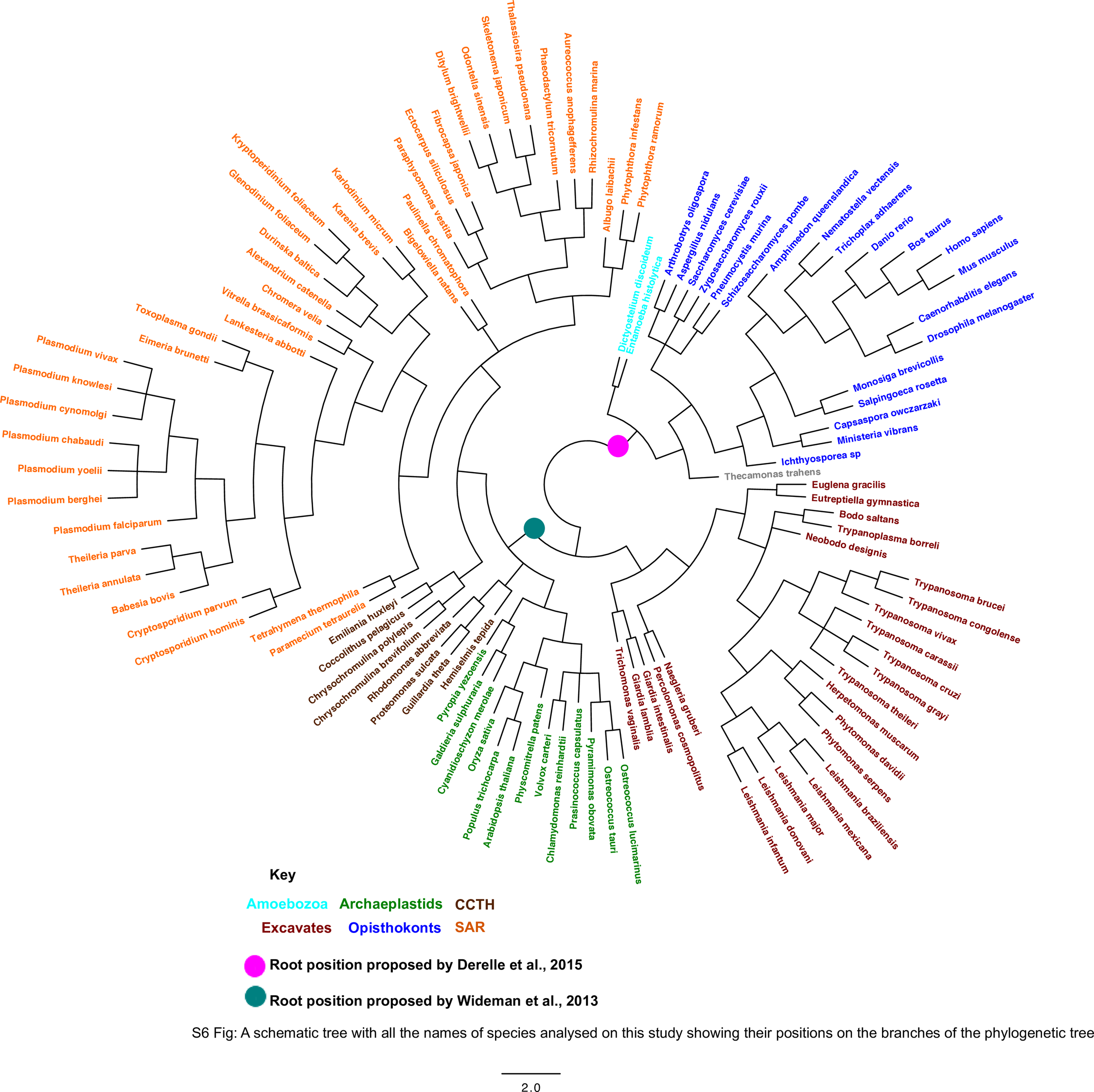

Supplement: S6 Fig — (TIF) [file pone.0192633.s006.tif]
